# Retraction: The role of individual level health resources on health outcomes of newly settled migrants in Sweden: Maissa Al-Adhami

**DOI:** 10.1093/eurpub/ckac184

**Published:** 2022-12-10

This is a retraction to: M Al-Adhami, E Berglund, J Wångdahl, K Hedrick, R Salari, The role of individual level health resources on health outcomes of newly settled migrants in Sweden: Maissa Al-Adhami, *European Journal of Public Health*, Volume 32, Issue Supplement_3, October 2022, ckac131.313, https://doi.org/10.1093/eurpub/ckac131.313

This abstract is being retracted as it was not presented at the conference and was included in the supplement erroneously.

